# Edward Tonkin Dobbins

**Published:** 1906-02

**Authors:** 


Edward Tonkin Dobbins, Esq., of Philadelphia, second vice-president of John Wyeth & Brother, incorporated, died at the University Hospital in that city, February 17, 1906, as the result of an injurry which occurred February 12. Mr. Dobbins fell on slippery pavement near his home, sustaining a fracture of the hip. Dr. J. William White was summoned and had the patient removed to the hospital. Mr. Dobbins, who was near seventy, did not rally from the shock of the injury, despite skilful efforts to overcome it, and gradually grew weaker until the end. Mr. Dobbins was an accomplished gentleman whose many friends will miss his genial presence.

